# A cost-effectiveness analysis of a residential radon remediation programme in the United Kingdom

**DOI:** 10.1038/sj.bjc.6690836

**Published:** 1999-12

**Authors:** C A Kennedy, A M Gray, A R Denman, P S Phillips

**Affiliations:** Health Economics Research Centre, Institute of Health Sciences, University of Oxford, Oxford, OX3 7LF, UK; Medical Physics Department, Northampton General Hospital, Cliftonville, NN1 5BD, UK; School of Environmental Science, University College Northampton, Park Campus, Boughton Green Road, Northampton, NN4 7AL, UK

**Keywords:** cost-effectiveness analysis, model, radon remediation, lung cancer, prevention, United Kingdom

## Abstract

As residential radon programmes of identification and remediation have proceeded, so questions have been raised about their costs and benefits. This study presents a generalizable model for estimating the cost-effectiveness of a radon mitigation programme using the methodological framework now considered appropriate in the economic evaluation of health interventions. Its use will help to inform future discussion of radon remediation and lung cancer prevention programmes. Data from Northamptonshire were analysed, resulting in a societal cost-effectiveness ratio of £13250 per life-year gained in 1997. The percentage of houses found to be over the action level, and the percentage of householders who decide to remediate are shown to be important parameters for the cost-effectiveness analysis. Questions are raised about the particular importance of perspective in this type of analysis and suggestions are made for future research directions. © 1999 Cancer Research Campaign


British Journal of Cancer (1999) **81**(7), 1243–1247
© 1999 Cancer Research Campaign
Article no. bjoc.1999.0836

### A cost-effectiveness analysis of a residential radon remediation programme in the United Kingdom

**CA Kennedy[1], AM Gray[1], AR Denman[2] and PS Phillips[3]**

[1]Health Economics Research Centre, Institute of Health Sciences, University of Oxford, Oxford OX3 7LF, UK; [2]Medical Physics Department, Northampton General Hospital, Cliftonville, Northampton NN1 5BD, UK; [3]School of Environmental Science, University College Northampton, Park Campus, Boughton Green Road, Northampton NN4 7AL, UK

**Summary** As residential radon programmes of identification and remediation have proceeded, so questions have been raised about their costs and benefits. This study presents a generalizable model for estimating the cost-effectiveness of a radon mitigation programme using the methodological framework now considered appropriate in the economic evaluation of health interventions. Its use will help to inform future discussion of radon remediation and lung cancer prevention programmes. Data from Northamptonshire were analysed, resulting in a societal cost-effectiveness ratio of £13250 per life-year gained in 1997. The percentage of houses found to be over the action level, and the percentage of householders who decide to remediate are shown to be important parameters for the cost-effectiveness analysis. Questions are raised about the particular importance of perspective in this type of analysis and suggestions are made for future research directions.
© 1999 Cancer Research Campaign

**Keywords:** cost-effectiveness analysis; model; radon remediation; lung cancer; prevention; United Kingdom

Radon is a naturally occurring radioactive gas that is emitted from certain types of rock. On average, radon contributes 50% of the natural background radiation dose to the general public (Hughes and O'Riordan, 1993). Radon usually dissipates harmlessly into the atmosphere but in affected areas it can reach concentrations up to 1000 times higher than natural background levels (Phillips and Denman, 1997) creating health hazards, specifically an increased risk of lung cancer (NRPB, 1993; Darby et al, 1998). It is possible to detect radon-affected buildings and make relatively simple alterations to deal with the problem. Such remediation measures include the installation of a gas-proof membrane or mat and improvements in ventilation.

In the UK, the National Radiological Protection Board (NRPB), an independent Government body, the Department of the Environment, Transport and the Regions, and local councils have in the past shared an interest in identifying affected buildings and encouraging remediation where radon exposure levels are over the UK domestic Action Level of 200 Bq $m^{-3}$. An area is classified by the NRPB as radon-affected if more than 1% of households are above the action level: these currently include parts of Cornwall and Devon, Derbyshire, the Mendips, Somerset, North Oxfordshire, Northamptonshire, Scotland, Northern Ireland and Wales.

As the programmes of identification and remediation have proceeded, so questions have been raised about their costs and benefits (Bradley and Thomas, 1996). Several published studies of radon detection and remediation programmes in domestic properties have reported only estimated and actual programme costs, expressed in costs per lung cancer case averted: for Spain (Colgan and Gutierrez, 1996), for Sweden (Snihs, 1992), for Canada (Létourneau et al, 1992), for the USA (Marcinowski and Napolitano, 1993) and for Northamptonshire (Denman and Phillips, 1998). Mossman and Sollitto (1991) estimated benefit/cost ratios of residential radon policy proposals in the USA, but did not include all direct costs (identification costs). No study to date has estimated the cost-effectiveness of a radon mitigation programme using the methodological framework now considered appropriate in the economic evaluation of health interventions (Gold et al, 1996; Drummond et al, 1997). It is important that this should be done, in order that the resources required to obtain health gain from radon mitigation can be systematically compared with equivalent data for other health interventions.

In this study a cost effectiveness analysis of radon mitigation in residential buildings was undertaken, using the best available national data and information from Northamptonshire on the costs and effectiveness of radon identification and remediation, and the costs and health impact of lung cancer cases. The overall model outlined in this study is generalizable to any radon-affected areas by applying the appropriate regional parameters. The results should help to inform future discussion of radon remediation and lung cancer prevention programmes (i.e. smoking cessation interventions).

### MATERIALS AND METHODS

The goal of the remediation programme was a reduction in radon exposure and corresponding decrease in the number of radon-induced lung cancer cases. Radon exposure was translated into lung cancer cases using lifetime risk estimates derived from relative risk estimates in published epidemiological data (ICRP, 1987; NRPB, 1993). Outcomes were defined in terms of the survival gain from averting radon-induced lung cancer cases, and

*Received 15 February 1999*
*Revised 4 May 1999*
*Accepted 4 May 1999*

*Correspondence to:* Christine Kennedy, Health Economics Research Centre, Institute of Health Sciences, University of Oxford, Oxford OX3 7LF, UK

expressed in life-years gained. The survival gain was estimated using life expectancy data from cancer registries (Cancer Intelligence Unit, 1992) and national life tables (ONS, 1991). The incremental cost-effectiveness of remediation programme versus no programme was then calculated as the ratio of net change in cost to net change in outcome.

The net cost of radon remediation was calculated by obtaining information on the cost of identifying households over the action level, the capital, maintenance and running costs of remedial work, and the treatment costs of lung cancer cases. The initial programme costs, including all measurement and remediation costs were assumed to have been incurred in 1 year (year 0). VAT was included in the costs as appropriate.

Different government agents and households potentially incur the costs of identification, remediation and cancer treatment. At the time this study was performed, detection costs were incurred by the NRPB and local councils, remediation and running costs were borne by the homeowner (no households in the data used received council radon remediation grants), and costs of lung cancer cases, associated with the diagnosis, staging, treatment and palliative care accrued to the National Health Service. The main perspective adopted in this analysis is societal, and does not consider the distribution of costs (and savings) between different agents. However, given the potential incentive effects of the distribution of costs on remediation decisions, a secondary analysis by payer is also presented.

Uncertainty surrounding the main parameters used in the study was handled by reporting standard deviations around treatment costs, and by varying key parameters within plausible bounds in sensitivity analyses. A one-way sensitivity analysis was undertaken. Probabilities surrounding uncertain parameters were not available to carry out probabilistic sensitivity analysis (Briggs et al, 1994).

All costs and outcomes were expressed in present values by applying a 6% annual discount rate to future costs and outcomes, as currently recommended in the UK (HM Treasury, 1997). Costs are expressed in 1997 prices. The time horizon for the valuation of costs and outcomes was 40 years, based on the anticipated life expectancy of the remediation and the mean manifestation period, where full expression of pulmonary malignancy after initial exposure to radon decay products is expected at 40 years (UNSCEAR, 1977).

### Data

### *Radon identification costs*

The unit cost of measuring radon levels per household is estimated as £35, based on the delivery, removal, reading and reporting from two track etch detectors in different rooms for 3 months (Kendall et al, 1994). For the purposes of this analysis, the total identification costs must be allocated to those homes in a surveyed area where remedial action is taken. Detailed information is available on 62 households who decided to take remedial action (48 of these formed the data used in Denman and Phillips (1998). It is known from a previous study in Devon and Cornwall that on average 11% of households found to be over the action level decide to remediate (Bradley and Thomas, 1996). It is also known that 6.3% of all homes measured in Northamptonshire were found to be over the action level (Bradley et al, 1997). Consequently we can infer that measurements were taken in 8947 households, and the total cost of identification is therefore £313 145. Follow-up detectors for those homes who decided to remediate, to assess the effectiveness of the remedial action, increases the total cost to £315 315. The VAT on the remediation costs is transferred to the Government, reducing the cost figure by £5777 to £309 538.

### *Remedial work*

All but two houses were remediated by at least one fan. The total capital cost of remedial work in the 62 households for which detailed information is available was £38 790 (including VAT). Maintenance and running costs, including electricity to run fans, spare parts and repairs and fan replacements every 10 years, were £21 036 (discounted at 6%). The total cost of remedial work was therefore £59 926.

### *Lung cancer treatment*

Published costings for treating lung cancer cases are rare: only one relating to the UK had been published at the time of this study (Sanderson et al, 1992), with another in press (Wolstenholme and Whynes, 1999). The former study reported unit costs for each element of lung cancer treatment, and the average cost per case for a sample of 196 patients treated at Southampton General Hospital around 1990. The latter study reports average costs per case for a sample of 253 patients diagnosed in 1993 and treated in the Trent Region.

Table 1 sets out the mean treatment costs for different types of lung cancer as estimated by these studies. In addition, the Table shows the results of applying the Southampton unit costs of treatment elements to the average pattern of treatment received by all 220 lung cancer cases diagnosed in 1996 in the Cherwell district of Oxfordshire and in the South Northamptonshire, Northampton, Daventry and Wellingborough districts of Northamptonshire (Cancer Intelligence Unit, 1998). All costs in the Table are shown in 1997 prices, adjusted where necessary using the combined HCSC index 1996/97–1990/91 = 29.76%, and assuming that diagnosis costs in Southampton were £100 (Sanderson et al, 1992).

It can be seen that the estimates do not vary widely between studies or cancer types. As current epidemiology is inadequate to discriminate between radon-induced small-cell and non-small-cell lung cancers, a combined cost estimate is calculated. The

**Table 1** Mean treatment costs for lung cancer derived from different studies

| Patient group | Mean cost per patient, 1997 | s.d. | Source |
|---|---|---|---|
| Combined | £6137 | Unknown | Sanderson et al 1992 |
| Combined | £6873 | Unknown | Unit costs from Sanderson et al applied to 220 patients in Northamptonshire and N. Oxfordshire |
| Non-small lung cancer | £6750 | 7341.88 | Wolstenholme and Whynes 1999 |
| Small lung cancer | £6199 | 4426.24 | Wolstenholme and Whynes 1999 |
| Combined | £6678 | 7105.30 | Weighted average[a] derived from Wolstenholme and Whynes 1999 |

[a]Using Whynes and Wolstenholme average costs multiplied by the share of each type of cancer in Northamptonshire and North Oxfordshire (86.8% NSCLC and 13.3% SCLC)

© 1999 Cancer Research Campaign

Northamptonshire combined estimate was chosen as it relates to the same geographical area as the remediation programme.

*Lung cancer risk from radon exposure*

The magnitude of the risk of lung cancer from residential radon exposure is currently debated. The NRPB currently estimates the lifetime risk of lung cancer per working level month (WLM) as $3.5 \times 10^{-4}$ for each year of exposure (ICRP, 1987; NRPB, 1993), where $1.26 \times 10^6$ Bq $m^{-3}$ h = 1 WLM (Kendall et al, 1994). This conversion figure is used in the current study. Studies are currently underway to pool European and North American indoor radon risk estimates for lung cancer, and a more precise and useful estimate of risk is expected to result from these analyses, which are expected in early 2000 (Darby et al, 1998).

*Life-years gained per lung cancer case*

Using the Oxford Cancer Registry, all cases of lung cancer (ICD code 162) diagnosed between 1989 and 1990 in the region including Northamptonshire and North Oxfordshire were identified, and data were extracted on survival rates by age and sex. The number of life-years lost per lung cancer death was then calculated using 1991 life tables (ONS 1996) to estimate remaining years of life expectancy in the general population. The average number of life-years lost due to premature mortality from lung cancer was estimated as 13.51 years per case. This is close to an estimate of 13.5 years by the ICRP (1991). It is greater than the estimate of 6.93 years per case for all neoplasms reported in 1980 data by Godfrey et al (1986), and also greater than the estimates for breast cancer over all stages (1.9–10.9 years) reported by Wolstenholme et al (1998). However, the figure of 13.51 years is plausible given the lower average age of death from lung cancer compared to other cancers (Cancer Intelligence Unit, 1992).

### RESULTS

### Costs

The net cost of the radon programme consists of initial, follow-up detectors minus VAT on remediation, at a cost of £309 538; remedial work totalling £59 826, minus averted costs of the 4.72 lung cancer cases of £32 441. The net cost, discounted at 6% per annum over 40 years, is therefore £336 923.

### Outcomes

The total number of occupants in the 62 homes undertaking remediation was 149, and the annual total per person reduction in radon achieved by the remediation work was $2.85 \times 10^8$ Bq $m^{-3}$ h (or 2.26 WLM), assuming that occupants spend 19.2 h per day in the home (see Kendall et al (1994), Appendix A). This total dose reduction translates into 0.118 cases of lung cancer averted per year, equivalent to 4.72 cases over 40 years. A total of 63.77 life-years were gained from these averted lung cancer cases. Assuming these to be equally distributed over the 40-year period of analysis, discounting this figure at a 6% rate equals 25.43 life-years.

### Cost-effectiveness

Combining the cost and outcome results reported above, the incremental cost per life-year gained of a residential radon remediation programme compared to no programme is £13 250.

**Table 2** Cost-effectiveness ratios by analytical perspective

| Perspective | CE ratio (£/per life-year gained) |
|---|---|
| Homeowners | £2353 |
| UK Government: | |
| NHS | Net saving |
| UK independent Government body: | |
| NRPB and VAT | £12 172 |
| NRPB and no VAT | £12 399 |
| Combined | £13 250 |

The cost-effectiveness of the programme alters depending on the analytical perspective adopted. Under the policy prevailing at the time the data were collected, homeowners were not responsible for the initial and post-remediation detection costs, but did have to decide whether to proceed with remediation and did have to bear the costs of any remedial work undertaken plus VAT. From their perspective, therefore, the cost per life-year gained is £2353. From the National Health Service (NHS) perspective, the intervention is cost-saving and provides a health gain, and therefore dominates the alternative of no remediation programme. From the NRPB perspective (not including VAT), the cost per life year gained is £12 399. The NRPB is an independent Government body, partly funded by the Department of Health and advises on all hazards of both ionizing and non-ionizing radiations. Consequently, for each party the cost-effectiveness is better than when a societal perspective is adopted. See Table 2 for a summary.

### Sensitivity analysis

A one-way sensitivity analysis was undertaken with the data. This involved varying each uncertain component of the evaluation individually, while keeping the baseline parameter values for all the other components the same. Figure 1 summarizes the results from the one-way sensitivity analysis of eight parameters and assumptions, showing the resulting cost-effectiveness ratio as the parameter values are reset at plausible maxima and minima.

It is clear that the cost-effectiveness ratio is most sensitive to changes in the percentage of homes over the action level in the area and to the percentage of households found to be over the action level who choose to remediate. The cost-effectiveness ratio is also sensitive to changes in life expectancy per lung cancer case and the given estimates of lifetime lung cancer risk to radon dose. Varying the discount rate applied to costs of maintenance and replacement has little effect on the results, but varying the baseline 6% discount rate applied to life-years gained does alter the cost-effectiveness ratio of the intervention to under £10 000 at a 0% rate and to approximately £32 000 at a 10% rate.

Varying the estimated cost of treating lung cancer or of remediation have little effect on the cost-effectiveness results. However, it is known that the average cost of remediation against radon is changing as new techniques are adopted, and reductions in average costs of remediation may increase the percentage of households choosing to remediate and thereby imply greater changes to the cost-effectiveness ratio (Bradley and Thomas, 1996).

### DISCUSSION

To date, no study has estimated the cost-effectiveness of a radon remediation programme using an appropriate methodological

*© 1999 Cancer Research Campaign*

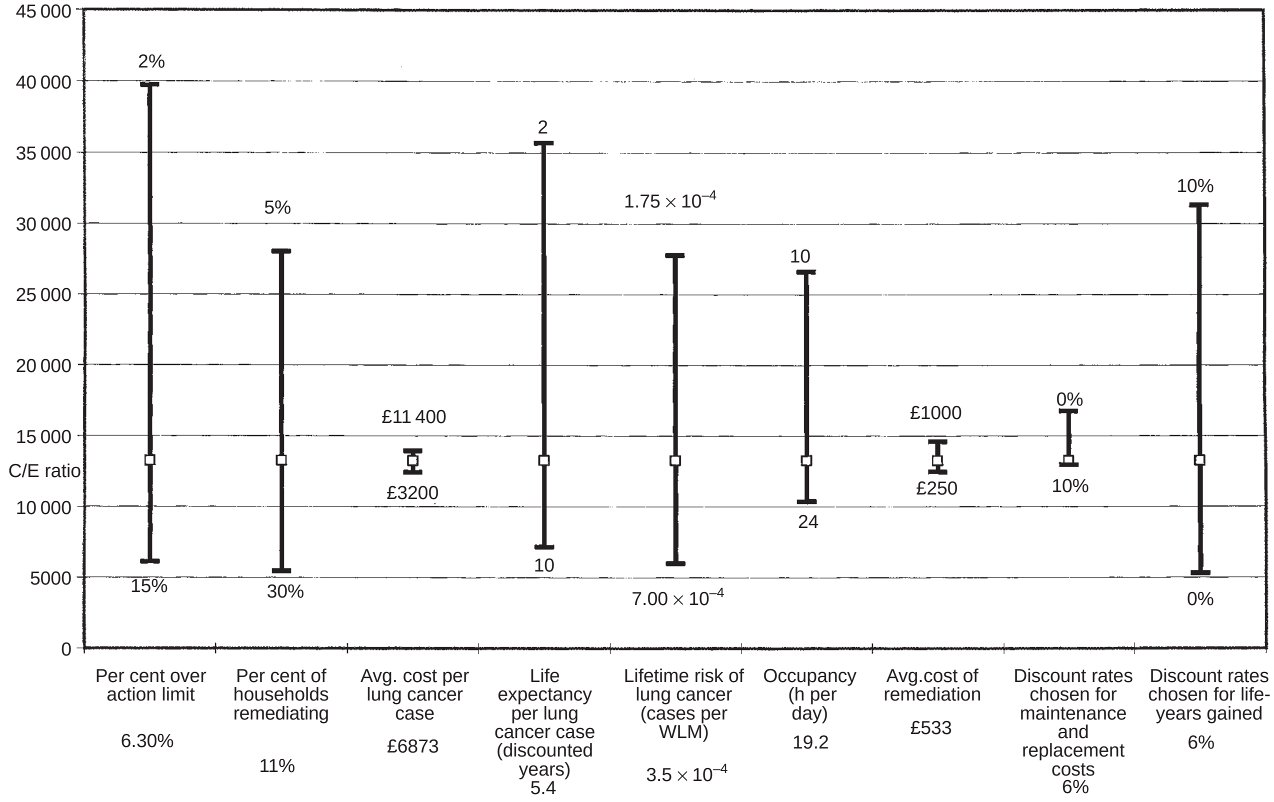

**Fig. 1** Sensitivity analysis framework for evaluating health interventions. In this study a model for the cost-effectiveness evaluation of a residential radon remediation programme was undertaken.

By evaluating the radon remediation programme in similar terms to other health interventions, comparisons can be made based on outcomes and costs per life year gained. When the cost-effectiveness ratio reported here for a residential radon remediation programme of £13 250 is compared to the results from all other cost-effectiveness ratios reported in published studies of health care interventions in the UK up to 1996 (they report all ratios, not only for interventions currently in practice), it falls between the sixth and seventh deciles and below the mean of £30376 (Briggs and Gray, 1999).

Assessing the cost-effectiveness of a particular intervention is complicated by the absence of any explicit 'ceiling' ratio in the UK health care sector. Prevention interventions which have been explicitly adopted or recommended for adoption – such as the breast cancer screening programme (Department of Health and Social Security, 1986) or secondary prevention of heart disease using statins (Johannesson et al, 1997) – have generally had cost-effectiveness ratios in the range of £5000–10 000 per life year gained, while interventions which have not been recommended lie in a range above £20 000–25 000 per life-year gained. However, many interventions currently in practice have cost-effectiveness ratios in excess of this range.

Such comparisons are difficult for two reasons. The first lies in the paucity of cost-effectiveness studies for lung cancer prevention programmes in the UK. Adherence to accepted methodological techniques in future evaluations of prevention programmes will facilitate comparison in the future. This also highlights the need for the evaluation of other residential radon-induced lung cancer prevention programmes in other countries using similar methodological techniques.

The second reason comes from the fact that there is a very complex interaction of perspectives in household radon remediation. In most health intervention evaluations in the UK the decision of whether or not to offer or proceed with an intervention is made from an NHS perspective. However, in residential radon remediation a large proportion of the prevention programme costs and decisions lie outside the direct influence of the NHS and fall on individual householders and other government agencies like the NRPB. Results from the sensitivity analysis indicate that, for policy purposes, the potential for improving the cost-effectiveness lies in influencing the decision of householders to remediate.

Compliance is an important consideration in radon remediation. Anecdotal evidence exists that some householders, once a radon sump fan has been installed, unplug the fan in order to save on the electricity costs involved. Such actions undermine the estimated effectiveness of the remediation over the 40-year period. There are currently no estimates available for the extent or spread of this practice, and further investigation should be undertaken.

Policy has recently changed in the Northamptonshire region so that the NRPB no longer offers free testing services; the homeowners are now responsible for the detection costs. Based on this analysis we expect a shift of a large portion of the costs onto the homeowner and will correspondingly affect the cost-effectiveness ratios estimated from the householder's perspective in the future.

This and subsequent planned analyses may help to answer several policy questions. As the largest potential for improving the cost-effectiveness of the programme involves increasing the

*© 1999 Cancer Research Campaign*

percentage of homes over the action level who choose to remediate, some cost-sharing between households and Government departments may result in a net welfare gain. There are considerations of possible public or charitable subsidies of remediation and testing to increase the remediation rate of homes which test over the action level. Once the price elasticities (price of remediation) for remediation are known it will be possible to calculate the optimal target thresholds for household remediation and cost-effectiveness ratios which result in a maximum social welfare gain. A study to estimate price elasticities for residential radon remediation programmes in the UK is underway; but, results are not expected until 2000. This information will be useful for informing future policy decisions.

### ACKNOWLEDGEMENT

The authors are grateful for the cooperation of Roger Tornberg of Radon Search (UK), Brixworth, Northampton in the collection of data.

### REFERENCES

Bradley EJ and Thomas JM (1996) *An Analysis of Responses to Radon Remediation Advice*. Chilton, NRPB-M707. TSO: London

Bradley EJ, Lomas PR, Green BMR and Smithard J (1997) *Radon in Dwellings in England 1997 Review*. Chilton, NRPB-R293. TSO: London

Briggs A, Sculpher M and Buxton M (1994) Uncertainty in the economic evaluation of health care technologies: the role of sensitivity analysis. *Health Econ* **3**: 95–104

Briggs A and Gray AM (1999) Handling uncertainty when performing evaluations of health care interventions. *Health Technology Assessment* (in press)

Cancer Intelligence Unit (1992) Record queries. CIU: Oxford

Cancer Intelligence Unit (1998) Record queries. CIU: Oxford

Colgan PA and Gutierrez J (1996) Cost effectiveness of reducing radon exposure in Spanish dwellings. *J Radiol Prot* **16**: 181–190

Darby S, Whitley E, Silcocks P, Thakrar B, Green M, Lomas P, Miles J, Reeves G, Fearn T and Doll R (1998) Risk of lung cancer associated with residential radon exposure in south-west England: a case–control study. *Br J Cancer* **78**: 394–408

Department of Health and Social Security (1986) *Breast Cancer Screening: Report of a Working Group (Sir Patrick Forrest, chairman) to the Ministers of England, Wales, Scotland and Northern Ireland*. HMSO: London

Denman AR and Phillips PS (1998) A review of the cost effectiveness of radon mitigation in domestic properties in Northamptonshire. *J Radiol Prot* **18**: 110–124

Drummond MF, Stoddart GL and Torrance GW (1997) *Methods for the Evaluation of Health Care Programmes*. OUP: Oxford

Godfrey C, Hardman G and Maynard A (1989) *Priorities for Health Promotion: An Economic Approach*. Discussion Paper 59. Centre for Health Economics: York

Gold MR, Siegel JE, Russell LB and Weinstein MC (1996) *Cost-Effectiveness in Health and Medicine*. OUP: New York

HM Treasury (1997) *Appraisal and Evaluation in Central Government*. The Stationary Office: London

Hughes JS and O'Riordan M (1993) *Radiation Exposure of the UK Population – 1993 Review*, National Radiological Protection Board, Report R263. NPRB: Chilton.

ICRP (1987) *Lung Cancer Risk from Indoor Exposures to Radon Daughters*. ICRP Publication 50. Ann ICRP **17**:

ICRP (1991) *Recommendations of the International Commission on Radiological Protection*. ICRP Publication 60. Ann ICRP **20**: 134

Johanesson M, Jonsson B, Kjekshus J, Ollsson AG, Pedersen TR and Wedel H (1997) Cost effectiveness of simvastatin treatment of lower cholesterol levels in patients with coronary heart disease. Scandinavian Simvastatin Survival Study Group. *N Engl J Med* **336**: 332–336

Kendall GM, Miles JCH, Cliff KD, Green BMR, Muirhead CR, Dixon DW, Lomas PR and Goodridge SM (1994) *Exposure to Radon in UK Dwellings*. Chilton, NRPB-R272. TSO: London

Letourneau EG, Krewski D, Zielinski JM and McGregor RG (1992) Cost Effectiveness of radon mitigation in Canada. *Radiat Prot Dosim* **45**: 593–598

Marcinowski F and Napolitano S (1993) Reducing risks from radon. *Air Waste* **43**: 955–962

Mossman KL and Sollitto MA (1991) Regulatory control of indoor Rn. *Health Phys* **50**: 169–176

NRPB (1993) Estimates of late radiation risks to the UK population. *Documents of the NRPB* **4**:

Phillips PS and Denman AR (1997) Radon: a human carcinogen. *Sci Prog* **80**: 317–336

Sanderson H, Mountney L and Harris J (1992) Cancer of the lung. In: *Health Care Needs Assessment*. NHS Executive: London

Snihs JO (1992) Swedish Radon Programme. *Radiat Prot Dosim* **42**: 177–184

United Nations Scientific Committee on the Effects of Atomic Radiation (1977) *Sources and Effects of Ionizing Radiation*. Report to the General Assembly, with Annexes. United Nations: New York.

Wolstenholme JL Smith SJ and Whynes DK (1998) The costs of treating breast cancer in the United Kingdom: implications for screening. *Intl J Technol Assess Health Care* **14**: 277–289

Wolstenholme JL and Whynes DK (1999) The hospital costs of treating lung cancer in the United Kingdom. *Br J Cancer* (in press)

© 1999 Cancer Research Campaign
